# Fair Shares and Sharing Fairly: A Survey of Public Views on Open Science, Informed Consent and Participatory Research in Biobanking

**DOI:** 10.1371/journal.pone.0129893

**Published:** 2015-07-08

**Authors:** Yann Joly, Gratien Dalpé, Derek So, Stanislav Birko

**Affiliations:** 1 Centre of Genomics and Policy, McGill University, Montreal, Quebec, Canada; 2 School of Public Health, University of Montreal, Montreal, Quebec, Canada; Centre for Geographic Medicine Research Coast, KENYA

## Abstract

**Context:**

Biobanks are important resources which enable large-scale genomic research with human samples and data, raising significant ethical concerns about how participants’ information is managed and shared. Three previous studies of the Canadian public’s opinion about these topics have been conducted. Building on those results, an online survey representing the first study of public perceptions about biobanking spanning all Canadian provinces was conducted. Specifically, this study examined qualitative views about biobank objectives, governance structure, control and ownership of samples and data, benefit sharing, consent practices and data sharing norms, as well as additional questions and ethical concerns expressed by the public.

**Results:**

Over half the respondents preferred to give a one-time general consent for the future sharing of their samples among researchers. Most expressed willingness for their data to be shared with the international scientific community rather than used by one or more Canadian institutions. Whereas more respondents indicated a preference for one-time general consent than any other model of consent, they constituted less than half of the total responses, revealing a lack of consensus among survey respondents regarding this question. Respondents identified biobank objectives, governance structure and accountability as the most important information to provide participants. Respondents’ concerns about biobanking generally centred around the control and ownership of biological samples and data, especially with respect to potential misuse by insurers, the government and other third parties. Although almost half the respondents suggested that these should be managed by the researchers’ institutions, results indicate that the public is interested in being well-informed about these projects and suggest the importance of increased involvement from participants. In conclusion, the study discusses the viability of several proposed models for informed consent, including e-governance, independent trustees and the use of exclusion clauses, in the context of these new findings about the views of the Canadian public.

## Introduction

According to the public Population Project in Genomics and Society, a biobank is “An organized collection of human biological material and associated information stored for one or more research purposes” [1]. These repositories are an increasingly important resource for the investigation of complex interactions between genetic and environmental factors in human health and disease [2]. Biobanks allow samples and biological data to be used repeatedly over time and are often linked to real-time changes in participants’ health, lifestyle, genealogical, and demographic data [2,3]. By categorizing these genomic and phenotypic variations, researchers can identify novel biomarkers useful for the development of personalized medicine [2,4]. To various extents, all public funding agencies endorse open science and data sharing policies as a way to accelerate biomedical research in public health [5,6]. These methods can also promote the goals of biobanking by fostering new research, enabling the exploration of topics not envisioned by the initial investigators and combining multiple sources to create new datasets [5].

Large-scale international biobanking can present ethical challenges regarding project governance and the respect of theoretical bioethical principles in research [7,8]. The protection of participants’ autonomy in biobanking, for example, is exercised through written informed consent procedures [8,9], an ethics requirement derived from the Nuremberg code [10] and reflected in international ethics texts [11,12]. Informed consent generally requires that participants be provided full information “about the nature of the research protocol” and all potential associated risks for every project involving the samples and data [13,14]. However, long-term storage and international open data sharing practices in biobanking may conflict with traditional informed consent norms, which require that specific information be provided concerning future research projects not foreseen at the time of the initial recruitment [15]. Therefore, these practices could prevent participants in biobanking projects from fully exercising their formal autonomy rights as traditionally defined [4].

Another issue arising from international biobank research involves the fact that laws governing the donation of genetic samples and data vary across jurisdictions. Even within a given country, the status of such materials and the identities of the individuals responsible for its safekeeping are not always clear. The words “control”, “custodianship” and “ownership” are at times used to describe the relationship between various stakeholders and the collected samples and data [16,17].

Novel consent models allow research participants in biobanking to exercise a greater degree of control over future use of their samples and data. For instance, the tiered consent model, also known as line-item or multilayered consent, asks participants to select from multiple options indicating their desire to be recontacted and the types of research in which their samples and information could be used. Another approach, called the selective, repeated, or reconsent model, requires that participants consent to each individual future study that wishes to use their data [18].

Nonetheless, it has been proposed that participants’ consent should depart from overly focusing on protecting their autonomy and should instead espouse a more communal view of research, embracing the values of altruism, solidarity and citizenry [7,8,19]. In donating data and samples to a biobank, research participants would agree to make them available to the research community nationally and internationally in the spirit of open science [5]. In turn, the awareness of resulting benefits such as improved knowledge, new therapeutics, diagnostics and research tools would increase participants’ trust in biobank and data-sharing projects [20]. One way of achieving this vision of genomic research might be to adopt an approach known as “broad consent”.

Broad consent grants researchers the permission to use samples and associated data for a broad range of research studies (such as the general categories of cancer research or behavioural research), typically including future and secondary uses unforeseen at the time of recruitment, subject to ethics oversight [4,18]. Unlike traditional, tiered or specific informed consent, broad consent could provide a pragmatic balance between protecting participants’ autonomy and achieving statistical significance through global data sharing [20,21]. Advocates of the broad consent model write that research participants who receive information from biobanks about research goals and potential health benefits can exercise an autonomous choice that is compatible with the nature of the science, their own personal values and informed consent requirements [7,8]. Their opponents object that “broad consent is not truly informed consent, but is rather a generic authorization that sacrifices the right of the donor to self-determination in favour of research interests” [15]. They argue that it does not respect the principle of patient autonomy by not adequately informing participants, in real time, of the specific nature, risks and benefits of the research in question [3,20,22]. A middle ground position considers broad consent to meet the criteria for informed consent if it respects each of the following three conditions: “(1) personal data are treated confidentially, (2) donors are guaranteed the right to effectively withdraw consent at any time in the process, and (3) new studies are approved by an ethics committee" [21,23]. The subject is still widely debated by scholars in the ethical, legal and social issues (ELSI) community [3,21,24].

Canadian legislation and jurisprudence require that consent obtained from research participants in biobank projects, like in any other research, be “informed”. Similarly, Canadian ethical guidelines demand that a sufficient amount of specific information about the nature and scope of the research be disclosed to research participants before their consent can be considered informed. On the other hand, funding agencies promote open science and international data-sharing for researchers using biobanks, and therefore indirectly encourage the adoption of broad consent practices. In response, it has been proposed that informed consent norms be reconceptualised to enable consistent and ethical broad consent practices in biobanks [4].

Public consultation enriches this debate by providing biobanks and policy-makers with a better understanding of participants’ concerns about key components of the informed consent process, such as the communication of information on future research projects, research governance structure, and the ownership of data and samples. Public consultation can also educate the public about the role and benefits of biobanks while enhancing public trust and participation in these projects [8]. Three studies of public perceptions on biobanking have previously been conducted in Canada; however, these were limited to the provinces of Quebec [25], British Colombia [26], and Alberta [27].

The first of these studies, by Godard et al. [25], focused on participation in Quebec’s CARTaGENE biobank. It began with a series of focus groups, which found that further information about issues such as confidentiality, transparency, return of results and personal benefit to participants would be needed for the interviewees to feel confident about participating in the biobank. These focus groups were followed up by a telephone survey, which found that both interest in and enthusiasm for this type of research were correlated with education level, and that those who had immediate experience of disease were most likely to be in favour. Men were more likely than women to be apprehensive of the CARTaGENE project, and the respondents' concerns primary involved confidentiality (34%), possible future uses of their information (26%), and a lack of information available (17%) [25].

The next study was conducted by O'Doherty & Burgess [26] in British Columbia. The authors used random dialing to recruit citizens from across the province and bring them together for two weekends in order to elicit general views, critical issues and biobank design recommendations. While they discovered a strong consensus in favour of biobanking and independent governance, there was less agreement on issues such as the process needed for researchers to gain access to samples and data; the ownership of samples and of any resulting pharmaceutical products; the distribution of benefits (including payment of sample providers); the use of other forms of consent such as broad consent, community consent, and consent for minors; sources of biobank funding; and forms of privacy protection. The discussion group reported that further discussion was needed on certain issues including those of ownership, patenting, benefits sharing and use of profits [26].

Caulfield et al. [27] performed a telephone survey finding significant variation in Albertans’ perceptions about biobanking. They asked respondents who they thought owned the samples involved in these projects, with 44% stating the institution, 26% the donor, and 23% the researcher. 58% preferred deidentification to anonymization, and 53% thought donors had a continuing right to make decisions regarding the use of their samples. 72% of Caulfield et al.’s respondents preferred that participants have the right to withdraw at any time. Although responses indicated that being recontacted for each new study would foster a sense of control, trust, and involvement, they also indicated a general opinion that being recontacted each time was both wasteful and bothersome. Indeed, 52% of respondents stated a preference for one-time consent, whereas 30% preferred tiered consent in the form of a list of options and 18% preferred to be recontacted each time. The authors found that these preferences for specific consent were correlated with those for continued control, return of incidental findings and overall distrust; they also found that men were generally more favourable towards research, while more educated individuals preferred broader consent and those with less education were more likely to believe that donors continued to own their samples. Finally, Caulfield et al. wrote that their study was limited by its geographical restriction to Alberta and by the lack of questions indicating a tradeoff between research and oversight [27].

The present study aims to incorporate the methods of these three studies in the development of a new exploratory survey spanning all Canadian provinces validating and complementing the findings of these previous studies. Therefore, the present study focuses on legal and ethical issues arising in three informed consent streams:

1. general information and consent structure
2. data control/ownership
3. governance of the infrastructure

In addition, this study assesses any further ethical concerns expressed by Canadians regarding participation in biobanks. It fills a number of gaps left by the three preceding studies. First, respondents from each Canadian province rather than a specific area completed the survey. Second, in recognition of the fact that Caulfield et al. asked their respondents who they thought owned samples, these findings are complemented in the present study by determining who respondents thought should ideally own them. The topics investigated also help fill O'Doherty & Burgess' call for further study on preferences regarding ownership, profit, and benefit sharing [26], while also making a contribution to our understanding of public opinion on the topic of consent. Furthermore, this study collects new quantitative data on some areas in which none seems to have previously been gathered in Canada, including opinions on the importance of receiving different types of information in the consent process, information sharing with the international community, and which stakeholders ought to take control and responsibility for the biobank's holdings.

## Methods

### Ethics Statement

Ethics approval for this study was given by McGill University’s Faculty of Medicine Research Ethics Board (study code A10-E81-12B). The informed consent documents, which contained information about the study “purpose”, “eligibility”, “risks and benefits”, “procedure and withdrawal” and “confidentiality”, were given as the online questionnaire’s cover pages. Respondents were informed that there were no foreseeable risks to them and that they could withdraw from the survey at any point. If they chose to participate, their answers could be published, but would be anonymized, with all uniquely identifying elements visible only to the authors of this paper and destroyed five years after the collection of the surveys. The respondents then gave informed consent by filling in checkboxes to indicate that they had read the consent form, were over 18 years old, and agreed to participate in the study (see questionnaire in S1 File).

### Study Design and Participant Recruitment

The inclusion criteria for participation in the survey were: being a resident of Canada; age 18 or over; ability to read English or French; completing all compulsory questions in the survey; self-identifying as being a past or potential future donor of tissue samples or genetic data to a biobank or genetic database (question 7 of the survey). This latter criterion was chosen in order to ensure that the selected individuals had sufficient interest and basic knowledge of biobanks to complete the survey, while also allowing the asking of questions about what the respondents thought should be done with respect to their own personal samples.

Three recruitment strategies were used so as to increase reach:

1. Printed advertisements for the survey were placed in the McGill University Health Centres (MUHC) affiliated hospitals.
2. A chain sampling method relying on 7 Canadian patient organizations, first contacted by email, who agreed to distribute the online survey to their members or subscribers using their own preferred means of communication. It was promised to the organizations that their involvement would not be made public for confidentiality purposes.
3. The research firm Ipsos [28] was hired to recruit additional respondents through their website www.i-say.com.

### Survey Development and Administration

The survey questionnaire consisted of 18 questions: six demographic questions, followed by twelve regarding the respondents’ perceptions and expectations of biobanking research and practices. The questionnaire was administered online using the paid service offered by SurveyMonkey.com [29], which is often used as a tool in academic research (see for example Wilson et al. [30]). The twelve questions regarding biobanking practice consisted of nine requisite multiple choice (MC) questions and three optional open-ended (OE) questions. These were divided into five sections by theme: “Informed Consent” (four MC and one OE questions); “Data and Sample Sharing” (two MC questions); “Control and Responsibility” (two MC questions); “Confidentiality” (one MC and one OE questions); and one concluding OE question. The full survey instrument can be seen in S1 File.

The first section on “Informed Consent” asked respondents to grade their perceived importance of receiving information regarding: project objectives; who would control the samples and data and who could profit from the biobanking research; the projects’ governance structure (including management and safeguards); and how their confidentiality would be protected. Respondents qualified their perceived importance of each type of information on a 4-point Likert scale of “very important”, “important”, “somewhat important”, and “unimportant”. In the second and third sections (“Data and Sample Sharing” and “Control and Responsibility”), respondents were given the opportunity to express their opinions on specific themes associated with biobanking ethics, specifically: the type of consent patients should give for the use of leftover tissue from medical procedures; whether biobank participants’ data should be shared at the local or international level; who should benefit financially from biobank research; and who should be legally and ethically responsible for samples and data. The fourth section on confidentiality included a question asking respondents to rate the importance they accorded to protecting the identity and medical records of biobanking research participants from access by third parties, using the same four-point Likert scale as described above.

The three optional open-ended questions encouraged the respondents to share their opinions concerning: what information should be provided about a biobank project in which they would consider participating; what risks they would associate with a breach of confidentiality in an open biobanking or genetic database research project; and what they foresaw, apart from consent and confidentiality, as additional issues or concerns about biobanking.

The survey opened on December 13, 2012.

### Data Analysis

The following four elements were designed as independent variables: province of residence; age; gender; and highest level of education completed. Correlations within independent variables as well as between dependent and independent variables were tested using the Pearson Chi-Square test on SPSS Statistics 22 [31] where the data satisfied assumptions relevant to said test [32, 33]. Tests were two-sided, and values of *p*<0.05 were considered statistically significant.

Thematic analysis of responses to the three open-ended questions was conducted independently by three researchers in the following manner. Each researcher read the responses and identified themes raised by the respondents, subsequently eliminating and grouping together sub-themes. There was, however, some overlap in the final themes classified by researchers. For example, several responses to question 18 were coded as both “insurance discrimination” and “use by corporations”. It was decided by consensus between the researchers to retain this level of overlap between categories in order to ensure that more general themes represented by specific examples were not overlooked. Nonsemantic responses were omitted from further thematic analysis. Each researcher independently labeled each response with the agreed upon relevant themes. The researchers then met and compared their analyses until no new themes emerged from discussion and consensus was achieved. Two researchers used Excel for coding and the third used NVivo [34]. When meeting to achieve consensus on coding, the researchers’ Excel table was used.

## Results

### Respondents’ Demographics

From February 2013 to July 2014, a total of 147 persons began the online survey. Response rate could not be calculated since chain sampling was used. Of the total 147 respondents, 114 (77.6%) satisfied the inclusion criteria and completed the online survey. Of the 114 respondents meeting inclusion criteria and completing the survey (RMICCS), 101 were recruited by Ipsos, 13 by the chain sampling method relying on Canadian patient organizations, and 0 via the advertisements placed in the MUHC affiliated hospitals. All 101 RMICCSs recruited by Ipsos completed the English questionnaire. Of the 13 RMICCSs recruited by patient organizations, 6 persons completed the questionnaire in French and 7 in English. A complete spreadsheet containing all deidentified responses to the questionnaire is available as S2 File.

Table 1 presents the RMICCSs’ demographics. The mean age was 42.16 years (median 42 years). Individuals from each of the ten Canadian provinces completed the survey (S5 File).

**Table 1:** Demographics of Survey Respondents.

Demographic Category	n	%
Overall	114	
Geographical Location within Canada		
West	30	26
Ontario	46	40
East	22	19
Do not wish to specify	16	14
Age, yrs		
18–34	38	33
35–49	39	34
50–74	36	32
Do not wish to specify	1	1
Gender		
Female	53	46
Male	61	54
Education		
Did not attend university	57	50
Attended university	56	49
Do not wish to specify	1	1

“West” includes the provinces of British Columbia, Alberta, Saskatchewan, and Manitoba. “East” is defined as Quebec, New Brunswick, Nova Scotia, Prince Edwards Island, and Newfoundland and Labrador. Respondents that selected that they had attended “some high school”, “graduated from high school”, had attended some or graduated from “college / CEGEP / trade school” were classified under “Did not attend university”. Those that self-identified as having attended “some university” or holding a “university undergraduate degree, such as a Master’s or PhD” or a “university graduate degree, such as a Master’s or PhD” were classified as “attended university”.

The mutual impacts between demographic variables are reported in Table 2. As no correlation in the table is significant at the *p*<0.05 level, the hypothesis that the demographic variables are distributed independently of each other is not rejected.

**Table 2:** Pearson Chi-Square of Demographic Variable Pairs.

	Geographical Location	Age	Gender
Age	1.324_4_		
Gender	3.67_2_	0.200_2_	
Education	0.409_2_	5.755_2_	0.319_1_

### Preferences for informed consent

Table 3 illustrates the importance respondents assign to receiving clear and specific information on key components of biobanking research.

**Table 3:** Importance of Receiving Clear and Specific Information on Key Consent Components and of Protecting Participant Confidentiality.

Component		Very important	Important	Somewhat important	Unimportant
Receiving information on:	Project objectives	80 [70.2%]	27 [23.7%]	5 [4.4%]	2 [1.8%]
	Governance structure	82 [71.9%]	22 [19.3%]	9 [7.9%]	1 [0.9%]
	Who would control samples and data and who could profit	80 [70.2%]	27 [23.7%]	6 [5.3%]	1 [0.9%]
	How confidentiality would be protected	84 [73.7%]	25 [21.9%]	4 [3.5%]	1 [0.9%]
Protecting identity and medical records from third parties		92 [80.7%]	17 [14.9%]	4 [3.5%]	1 [0.9%]

91 out of 114 RMICCSs responded to the open-ended question “What is the most important piece of information you would like to be provided about any biobank project that you participate in?” with an average response length of 12.9 words. Table 4 reports the most commonly identified themes in these responses, while the table of all themes can be seen in Table A in S3 File.

**Table 4:** Top 10 Themes in Respondents’ Desired Information about Biobank Projects.

Themes	n	%
project objectives	43	47
confidentiality	19	21
data protection	18	20
data management	15	16
return of results	13	14
relevance to donor	9	10
health applications	8	9
who benefits	7	8
use of profits	6	7
who has access	6	7

43 respondents answered that obtaining information on project objectives was most important, more than twice as many as selected the next most-common response (Table 4). One 58-year-old female respondent wrote that she would be most interested in hearing about “The specific method in which the sample would be used, for what purpose, the nature of the project being worked on, and how it is hoped that results would benefit medical science” Meanwhile, a 37-year-old female respondent favoured hearing about “the purpose and use of my samples and what will be done with my samples at the completion of the project”.

The second most common topic mentioned in response to this question was confidentiality, which was addressed by 19 respondents. For instance, a 35-year-old male respondent was more concerned about “How it is being used, how I am protected from corrupt or evil activities, and what precautions are taken to protect it”.

### Preferences for data and sample sharing

**Fig 1:**
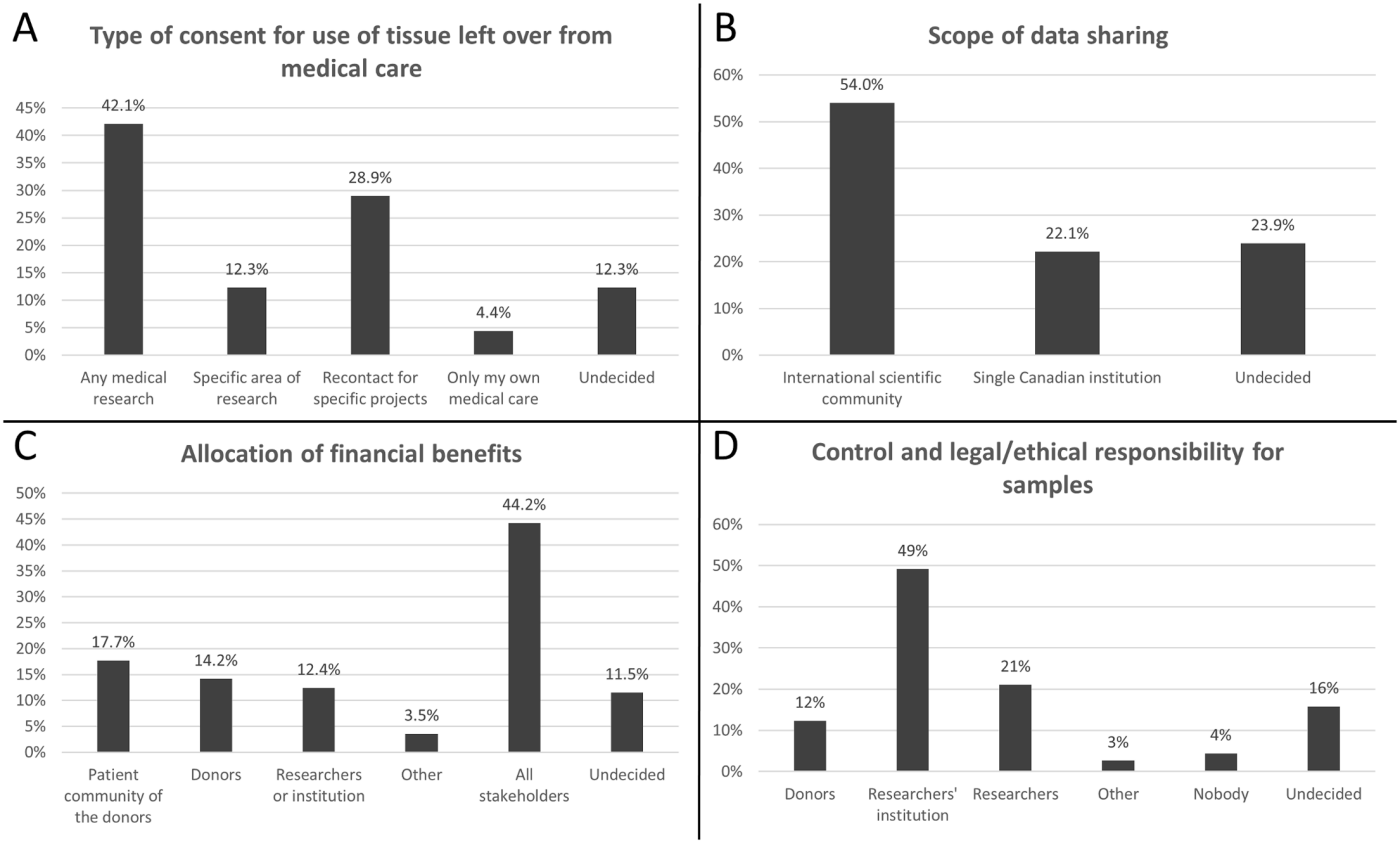
Preferences for consent models, data sharing, allocation of profits and ownership of samples and data. Respondents selected from among the listed options their preferred type of consent (A), scope of data sharing (B), use of any profits from the research (C), control over samples and data (D). All graphics display the percentages of participants opting for provided multiple choices. In order to reflect the fact that some participants used the “other” box to select more than one of the listed options, those answers were reassigned to the categories in question, resulting in percentages summing to over 100% in (C) and (D).

When asked what they would “prefer concerning tissue samples from your body if they are left over after a medical procedure”, 42% of the respondents (n = 48) preferred to provide a one-time general consent (Fig 1A). More respondents opted for more specific or restrictive forms of consent. These included consent to use their leftover tissues in a specific area of research (e.g. cancer research or genetic research) (12%, n = 14) and being re-contacted to give consent for each specific research project (29%, n = 33) (Fig 1A). Additionally, a few respondents preferred that their leftover tissues not be used except for their own medical care (4%, n = 5) (Fig 1A). Although it was the most common specific choice at 42%, survey respondents were not in consensus on the one-time consent for all future studies which is a key component of broad consent, showing that many respondents still prefer consent models limiting, to some extent, the future use of their samples and data. However, the provision of general consent authorizing research limited to a specific area (for example, research in cancer genetics) is considered a part of the broad consent category by some authors [18]. Considering this more flexible definition and combining the 14 latter results with the 48 choices for general consent, 54% of respondents were thus in favour of broad consent (n = 62) (Fig 1A).

Preferences of respondents self-identifying as female or male differed significantly, Χ^2^(4, N = 114) = 13.904, *p* = .008. Whereas female respondents (n = 21, 40%) preferred being re-contacted to give consent for each specific research project, males (n = 30, 49%) preferred one-time general consent. Additionally, responses to this question differed by level of education, Χ^2^(4, N = 113) = 9.554, *p* = .049. Respondents that attended at least some university preferred being re-contacted to give consent for each specific research project (n = 20, 36%), and those that attended no university preferred one-time general consent (n = 30, 53%). It is noted that no statistically significant dependence between gender and level of education was found in the study’s cohort (*p* = .319). Table 5 displays the responses to the four questions that assessed respondents’ preferences for different biobanking models (consent, scope of sharing, profit allocation, ethical and legal responsibility) according to the demographic categories of gender, age, education level, and geographic location within Canada.

**Table 5:** Data and Sample Sharing Model Preferences versus Demographic Characteristics of Survey Respondents.

What would you prefer concerning tissue samples from your body if they are left over after a medical procedure?							
	Only my own medical care	Any medical research	Specific area of research	Recontact for specific projects	Undecided		P value
F : M ratio	1:4	18:30	3:11	21:12	10:4		.008
Age (y)†	40±15.7 (20–59)	43±14.6 (19–73)	35±13.5 (21–69)	45±14.5 (22–73)	42±13.4 (26–73)		.169
University : No university ratio	5:0	18:30	7:7	20:13	6:7		.049
West : ON: East ratio	2:3:0	9:23:11	3:6:0	11:10:9	5:4:2		.191
Would you be willing to participate in a project where your information and samples would be shared with the international community (without your name or personally identifying information attached), or would you prefer to participate in a project in which your data was only used by a single Canadian institution?							
	Int’l scientific community	Single Canadian institution	Undecided				P value
F: M ratio	25:36	13:12	15:12				.381
Age (y)†	42±14.6 (20–73)	43±13.6 (19–66)	43±15.8 (23–73)				.145
University: No university ratio	32:29	12:13	12:14				.843
West: ON: East ratio	16:29:10	8:7:8	6:9:4				.418
Who should benefit financially from large-scale biobank research using genetic data and samples?							
	Donors	Patient community of the donors	Researchers or institution	All stakeholders	Undecided	Other	P value
F: M ratio	7:6	15:4	1:6	15:35	8:5	6:5	.003
Age (y)†	34.5±13.7 (19–59)	43±12.3 (24–61)	36±12.4 (22–54)	43±14.5 (20–73)	44±17.6 (25–73)	45±11.0 (22–69)	.886
University: No university ratio	5:8	9:10	4:3	25:25	5:7	8:3	.624
West: ON: East ratio	0:6:5	7:7:2	1:2:2	14:23:8	4:4:1	4:3:4	n/a*
Who should control and take ethical and legal responsibility for the genetic samples and data in a large-scale biobank or genetic database project?							
	Donors	Researchers	Researchers’ institution	Nobody	Undecided	Other	P value
F: M ratio	3:10	5:16	30:22	2:3	12:6	1:4	.013
Age (y)†	39±16.0 (19–73)	46±15.0 (22–69)	43±13.8 (20–73)	39±9.4 (24–53)	37±15.1 (21–73)	48±12.8 (30–63)	.169
University: No university ratio	4:9	13:8	25:27	5:0	7:10	2:3	.068
West: ON: East ratio	2:5:4	9:5:4	14:25:10	2:0:0	2:9:3	1:2:1	.185

^†^Data are: mean±SD (range)

*The assumptions for the Chi Square test used were not satisfied [32, 33].

Respondents’ perceptions of different models of consent further varied when asked about their preferences for international or local sharing of their data and samples. We introduced the next question by writing,

“Open sharing of genetic information between researchers and institutions has been shown to substantially facilitate health research. However, some parties are concerned that this could limit patients’ rights to choose how, and by whom, their genetic information is used.”

A majority of survey respondents reported they would be willing to “participate in a project where your information and samples would be shared with the international community (without your name or personally identifying information attached)”, provided the samples and data did not contain any personal identifiers (54%, n = 61) (Fig 1B). Of the 48 respondents that opted for one-time general consent in Fig 1A, 67% (n = 32) also opted for sharing their data and samples internationally. Although there is evidence of interest in broad sharing practices among that subgroup, it constitutes only 28% of the entire cohort and does not suggest that the public is in general agreement regarding all aspects of open science. On the other hand, we found that nearly half the survey cohort (46%) either preferred to participate “in a project in which your data was only used by a single Canadian institution” (22%, n = 25) or remained undecided on this question (24%, n = 27) (Fig 1B).

### Preferences for control and responsibility over samples and data

Financial benefits may result from biobanking activities through commercialization and/or property rights over the stored biological material and data. When asked “Who should benefit financially from large-scale biobank research using genetic data and samples?”, a large proportion (44%, n = 50) of the study cohort selected that “everybody” (Fig 1C) should benefit, including the listed categories of research participants, researchers and the general communities to which the participants belong. Respondents with more specific choices selected that financial benefits should accrue to the patient community to which the original donors belonged (17%, n = 19) the donors themselves (14%, n = 16), and the researchers conducting the project (11%, n = 13). A minority of respondents answered “no opinion” (11%, n = 13) or “other stakeholders” (10%, n = 11) to this question. Stated preferences differed by gender, Χ^2^(5, N = 113) = 18.199, *p* = .003 (Table 5). For the purpose of visualizing more clearly the respondents’ preferences, responses written in the “other” box were reassigned to the other categories if the respondent had used the open-ended answer option merely to select multiple of the listed categories.

A 49-year-old male respondent wrote that benefits should accrue to “The community for which the research is done. Whether or not the donor has that particular condition”. Several others wrote that nobody should reap financial benefits from this sort of research. One 35-year-old female respondent also wrote that: “if this material is meant to be used... for profit from drug companies, I would be very upset, as I don't think that is a very ethical use of biobanking / genetic database research.” Another 33-year-old female respondent wrote:

“I don't believe that organizations should profit from the sharing or collaboration on the use of this type of research. So there is a concern that for profit companies or even government institutions will take advantage of openly shared information for their own personal profit.”

Furthermore, a 44-year-old male respondent wrote that “the sample is given freely and the results should be freely given”. While this attitude suggests an opposition to commercialization in addition to the mere distribution of profits, it also indicates that many laypersons have principles in common with those of open science.

In order to gain a different insight into respondents’ beliefs on data and tissue ownership/custodianship issues, they were asked “Who should control and take ethical and legal responsibility for the genetic samples and data in a large-scale biobank or genetic database project?” The results show that nearly half of survey respondents (46%, n = 52) trust the researcher’s institution to take care and manage their information and samples as opposed to the researchers themselves (18%, n = 21) or the original data donors (11%, n = 13) (Fig 1D). 25% of respondents, however, responded “nobody” (4%, n = 5), “others” (4%, n = 5) or “undecided” (16%, n = 18) to this question. As with the previous question of this type, “other” answers merely listing multiple of the listed categories were regrouped into those categories prior to data analysis.

While responses to the question regarding ethical and legal responsibility followed different distributions depending on the respondent’s self-reported gender, Χ^2^(4, N = 109) = 12.759, *p* = .013, respondents from both categories preferred that “the institution of the researchers who conduct the biobank project” be in control and take responsibility. The biggest difference between the two groups was that the second most common preference among females was “undecided” (n = 12, 23%), and among males it was “researchers who conducted the biobank project” (n = 16, 26%), although the third most common preference among females was this latter response as well (n = 5, 9%).

### Preferences for confidentiality

In the section on confidentiality, 81% of respondents selected that it was “very important” for biobanks to protect participants’ identities and medical records from third parties (Table 3). Indeed, 19 of the 91 answers to the open-ended question on desired information expressed interest in learning about the confidentiality of their data, while 18 had asked about how it would be protected from third parties, respectively marking the second and third most common responses. The question of how data would be managed was the next most common with 15 responses (Table 4). For example, a 19-year-old female respondent wrote that she would expect to be informed “who will be responsible for managing my information and what safeguards are in place to protect the research participants”. Other fairly common responses to that question included 13 requests for information about return of results to the project participants, 9 about the project’s potential relevance to the participants personally, and 8 about its health applications (Table 4).

This was followed up with a second open-ended question asking specifically “What risks, if any, would you associate with a breach of confidentiality in an open biobanking or genetic database research project?” (Table 6).

**Table 6:** Top 10 Themes in Risks Respondents Identified with a Confidentiality Breach.

Themes	n	%
use by third party	39	60
identification of donor	35	54
use by corporations	17	26
insurance discrimination	16	25
disclosure of genetic risks	11	17
disclosure of personal information	10	15
identity theft	9	14
use in other research	8	12
employment discrimination	6	9
information released to general public	6	9

65 of 114 respondents responded to the question on confidentiality concerns, with an average response length of 17.7 words. The full table is available as Table B in S3 File.

The overarching concerns expressed by respondents involved use by third parties (n = 39) and the identification of specific donors (n = 35), with the most common specific risk mentioned being genetic discrimination in insurance (n = 16), followed by “identity theft” (n = 9) and discrimination in employment (n = 6) (Table 6). For example, a 53-year-old male respondent wrote that:

“Insurance companies, employers—private and governmental—could possibly gain access to and use this genetic material to search for otherwise undetectable risks for future illness. Which they could use to deny service, employment, pensions etc…”

In a similar vein, one 19-year-old female respondent wrote that “People may not want to disclose to family members the results of their genetics tests because of potential discrimination by insurance companies and concerns that test results may make the family uninsurable.” It is telling that many of our respondents identified insurance discrimination as an issue, given that this is one of the more hotly-debated ethical issues raised by genetics in Canada, yet no prompting on the topic was provided.

A 57-year-old female respondent summarized a further three issues, stating she would be concerned that “family or friends of those that contributed may be morally offended or hurt if names and information is breached [or the] Donor is made a subject of criticism by public/media etc. [or] Research is inhibited from moving forward.” Meanwhile, a 37-year-old female respondent wrote that “Unwanted contact from a third party for any reason with regard to the information they received as a result of the breach.”

Finally, a 65-year-old female respondent wrote that “I would not want to be another Henrietta Lacks”, referencing the increasingly well-known use of “HeLa” cells in decades of biomedical research before the original donor’s family was notified [35].

The final question of the survey asked participants “Other than the topics discussed above, do you foresee any additional issues or concerns that open, collaborative biobanking or genetic database research could create?” 30 of 114 respondents responded, with an average response length of 23.7 words. Due to the limited number of shared coding categories and substantial overlap with prior open-answer questions, the resulting table is presented in S4 File.

## Discussion

The present study investigated key elements of informed consent for biobank projects in order to better understand their relative importance in the eyes of individuals familiar with biobanking. It also assessed respondents’ desired level of involvement in the governance process of biobank projects. The findings provide biobank researchers, ethicists and policymakers with important information on the preferences of potential research participants that can be used to improve the informed consent process in the future. Such findings are timely given the current debate on the validity of broad consent and the interests of the research community in developing new, more participatory consent models [4].

### Types of consent

In general, respondents wished to receive clear and specific information about biobank project objectives, and expressed interest in being well informed about biobank projects in which they participate. These findings suggest that being better informed could benefit participants and boost their trust in data custodians or other responsible entities. Moreover, the present study’s findings may indicate that respondents’ desire for involvement in the use of their own data and samples might influence opinions on the type of consent participants prefer. Previous surveys in Europe confirm this trend [36].

As observed in other surveys [3,7,22], however, their opinions are divided about the type of consent they feel is most appropriate regarding donation of leftover tissue samples following a medical procedure. Like O’Doherty and Burgess [26], we found that no clear majority consensus seemed to exist with respect to a preference for types of consent, ownership of samples or distribution of benefits. Although respondents to this survey most commonly preferred broad consent and international data sharing, a significant proportion of respondents preferred restricting the use of their tissues toward specific research projects or specific areas of research. Whereas Caulfield et al. [27] found that 52% of Albertan respondents preferred one-time, 30% preferred to selected from a list of options, and 18% preferred to be contacted each time, our survey found that 42% preferred a one-time consent, 12% preferred to choose an area of research, and 29% preferred to be recontacted for each specific project.

A statistically significant difference was found between men and women, with the results indicating that men preferred one-time consent whereas women preferred being recontacted for each new project (p = 0.008). However, Godard et al. [25] had previously found that men from Quebec were more likely to be apprehensive of biobanking. The present study’s results are more similar to those of Caulfield et al. (2012), which found conversely that Albertan men were generally more favourable than women towards biobank research.

Similarly to both Godard et al [25] and Caulfield et al. [27], this study found that education was associated with different attitudes towards research, specifically towards consent models. Yet unlike Caulfield et al., who reported that higher levels of education was associated with preferences for broader consent, these results indicate that more educated individuals are more likely to prefer being recontacted to provide consent for specific research projects.

Interestingly, nearly half the respondents remained undecided or expressed a preference for restricting the usage of their data to the single institution to which they had donated their samples and information, highlighting a lack of consensus on both the scope of sharing and preferred type of consent. Combined with the high degree of importance they attribute to obtaining clear and specific information about research projects, this indicates that a significant fraction of research participants may be uncomfortable with broad consent unless given the opportunity to be more actively involved in the biobank governance structure in exchange for leaving greater freedom to the researchers.

### Biobank governance structures and control of data

The analysis of qualitative, open-ended answers from our survey revealed that many respondents were concerned over the potential use of their data by insurers, the government and other third parties outside the therapeutic or health research context. Moreover, quantitative answers confirm that participants expect to obtain clear and specific information about the governance framework and accountability rules of the biobank regarding their samples and data. This information could increase their willingness to participate in biobank research while providing the biobanks and research institutions with greater control of their samples and data. Some authors [24,37] have suggested that the public should be considered a more “integral part of the overall governance structure of the biobank” [38]. Indeed, a substantial number of respondents preferred the ability to remain involved in future decisions that implicate their data and samples. The results of the present study therefore support the findings of other studies suggesting that participants want to have a more participative role in research, including decisions regarding the use of their samples and data [22,39].

This study found that respondents preferred assigning control as well as legal and ethical responsibility for samples and data to the researcher’s institution rather than keeping control themselves or giving it to the researchers collecting the data. This is an important finding in opposition to the recommendation of certain legal scholars that research participants be given ownership rights over their genetic material and data. Granting such “personal” ownership could actually preclude greater participant involvement in biobanking projects, as they would likely need to spend a substantial amount of time negotiating complex contractual arrangements in addition to already lengthy consent forms [22,24].

### Financial benefits of biobank research

The private sector increasingly contributes to biobanking activities through public-private partnerships [40]. Given the substantial cost of creating and maintaining a biobank, steady financial support is essential for the survival of these projects. The need to secure private funding can be difficult to reconcile with the increasing pressure biobanks face to facilitate open science through unrestricted data sharing. If the possibility of using conventional business strategies such as secrecy or intellectual property to ensure a return on investment is curtailed by open data sharing, commercial interest in financing a biobank could be significantly reduced [40,41].

When asked who should benefit financially, a majority of respondents were in agreement with a more democratic sharing model favouring all stakeholders. The scientific, humanitarian and clinical contributions of biobanks [9,38,39], presented to the participants through regularly updated public information on the progress of the researches, could be considered public goods from which both research participants and the community at large would derive benefits.

Furthermore, qualitative answers show that respondents attribute a high degree of importance to receiving clear and specific information about who should profit financially from biobank and genetic database projects. It is possible that research participants would better understand the need to recoup investments in these projects if more information about the high financial and human-resource costs involved in biobank development and maintenance were more clearly communicated to them.

### Novel approaches to informed consent

The lack of a clear consensus about the most appropriate type of consent approach in our survey could be explained by the different types of relationships that participants might have with a biobank, the scale of the biobank and the socio-cultural particulars of various participant communities [20,42–44]. Thus, policymakers and researchers should not necessarily promote a one-size-fits-all type of consent. They may prefer to develop more tailored consent approaches that consider, in addition to the role of the biobank, the experiences, perceptions and expectations of different participants and participant communities [45]. Our survey respondents displayed an interest in broad consent practices that would nevertheless enable them to retain some degree of control over secondary use of their data and samples, as well as provide them with sufficient and clear information on applicable governance rules. Given the popularity of the broad consent approach in the research community and the challenge of reconciling open-science research with traditional consent, novel approaches that afford the participant a more active, participatory role in the governance structure of biobank projects should be considered [7].

Some authors who share this view have proposed adding ‘exclusion clauses’ to biobank participants’ consent forms [44]. These would permit participants to indicate certain research areas they consider contentious, and for which they would not consent to the use of their data and samples. This would partially address concerns of social risks if participants were to be associated with projects they perceive as particularly stigmatizing, such as HIV, schizophrenia, race/ancestry determination, inbreeding, alcoholism, and sexual orientation research [18]. Such clauses would be easily included in consent forms irrespective of the preferred consent model (e.g. broad, tiered, or specific), and could facilitate more democratic governance and greater accountability of biobanks through greater transparency in the use of future samples. However, allowing participants to select research projects in this way might make it increasingly difficult to develop and conduct research projects in certain important, yet sensitive, medical areas, and would certainly complicate international data sharing by imposing reach through research limitations to downstream data issues. Most biobank developers surveyed on this point nevertheless feel exclusion clauses could be a good compromise to foster trust among researchers, biobank developers and participants, and could result in increased participation in these projects [18].

Exclusion clauses are written by researchers rather than participants, and as such, laypersons might have some difficulty determining their content [18]. Public surveys like those used in this study might provide suggestions about what participants consider contentious practices or areas of research, such as patenting, race-related research, recontact by third parties, and human cloning (Table 6). While exclusion clauses would likely function well with small biobanks, genetic data derived from these biobanks could be difficult to integrate into a larger-size repository given the need for the initial researcher to respect these consent limitations [18]. In the context of broad consent and open science policy, there is a need to allow expansion of biobanks without the administrative burden of continually attempting to recontact participants. When using exclusion clauses, researchers would need to foresee which research topics participants could exclude without hindering future collaboration with other biobanks or contravening their funding guidelines.

E-governance is a model recently proposed for large-scale, public, population-based biobanks as a way to circumvent problems associated with small biobank expansion, including the perceived top-down governance structure, and to ensure more participation from all stakeholders [46]. E-governance represents a “Web 2.0” digital forum, meaning that any registered biobank contributor could collaborate online by proposing, drafting and amending proposals for governance structures, protocols, strategy and policies. Adapted to the reality of large-scale population biobanks, such a governance structure would best incorporate ideas from the research community while giving a voice to participants and allowing them to identify their own concerns (a goal which could be accomplished, for example, through surveys). This collaborative community effort would ensure greater buy-in to biobank governance structure and policies, since they would be crafted in partnership with the participants rather than imposed [46]. Furthermore, the e-governance model borrows from the open science model, facilitating collaborative efforts from a broader base of stakeholders. Nonetheless, some participants and researchers might see such a participatory system as overly complex, abstract, or time-consuming [45].

Respondents were generally willing to delegate control of their data and samples to a custodian (e.g., the researchers’ institution). Some scholars who recommend this model have proposed using advisory groups as mechanisms for representing the views of a community of participants and re-assessing the nature of the initial consent in any situation of potential conflicting interest [8]. Winickoff proposes that biobank participants transfer their property interest in data and samples to a “charitable trust”, a trustee who as a legal fiduciary would keep or use the property rights for the benefit of the designated group [47]. This legal entity would be compatible with the altruistic nature of biobank donation for the benefit of the public, could act in an advisory role for the participant group, and might provide greater stability over time with regards to samples and data as opposed to the possible bankruptcy risks of private biobanks [47,48]. As Hanson points out, bankruptcies could pose a particular ethical issue since some jurisdictions allow tissue banks to be sold with few restrictions, with one human cell collection in Japan having been auctioned to the highest bidder [48]. The “charitable trust” model could also increase trust in the biobanking research enterprise and enhance participation [47]. The administrative burden of research associated with a more widespread use of this “charitable trust model” is, however, not documented.

### Limitations

The study was limited, at first, by the fact one of our methods did not secure any respondents whatsoever, whereas the second only produced 13. Thus, the hiring of a private research company to recruit respondents proved imperative, which meant that sample size was limited by budgetary considerations. The final sample size of 114 respondents is not statistically sufficient to represent the Canadian adult population as a whole. However, statistical representation was not an original aim of the study, and since the final study cohort spanned all provinces, age groups, genders, and education levels, it was deemed to provide diversity sufficient for the purposes of the study. On the other hand, the study originally aimed to compare past donors and non-donors’ interest in taking part in biobanking projects, but the final amount of donors (n = 21) was not sufficient for statistical analysis.

The study was also limited by the fact that not all demographic data was provided by all respondents. For example, specific mailing addresses were not required from respondents for privacy purposes. Although most did list their locations, the missing data prevented consideration of every respondent when comparing responses between provinces of Canada. A further limitation is general to all surveys, in that there is always a risk of participants misinterpreting questions and not signalling it to the researchers. Finally, we only recruited respondents who were potentially interested in participating, or had already participated in biobank projects. This likely introduced a bias favourable to biobank research in our results. This selection criterion was essential for our study, however, in that it allowed us to target the actual members of the general population who had sufficient interest in our survey and a basic knowledge of biobank research.

## Conclusions

This study focused on exploring the opinions of previous and potential research participants regarding a number of consent-related themes in biobank research. As a secondary objective, it aimed to identify additional ethical issues that might concern Canadians interested in participating in genetic biobank projects.

The respondents generally favoured broad consent practices that permit international sharing of research data and samples. Despite a strong preference for broad consent practices, this survey corroborates other findings that indicate a lack of consensus regarding the most appropriate consent model to promote participants’ right to autonomy in biobank research. Rather, a variety of perspectives is highlighted, some of which cannot be accommodated through broad consent. A significant number of respondents preferred reconsent, specific consent and only local sharing of data, all of which are more compatible with traditional informed consent practices. Their concerns regarding data control and project governance indicate that our respondents desired more involvement in the oversight of the biobank as a whole. These findings suggest that current consent models such as traditional or broad consent alone do not completely address participants’ expectations.

It has been proposed that this divergence of opinion could result from not sufficiently accounting for the variety of interests among stakeholders, different types of biobanks and diverse participant communities [45]. We propose that using a variety of more participatory approaches such as integrating exclusion clauses into consent forms, using e-governance models, or transferring ownership of the data and samples to an independent trustee could foster increased participation in biobanks [7] and contribute to a more democratic, international and productive biomedical research process that would also be respectful of patients’ autonomy and dignity.

## Supporting Information

S1 File Questionnaire: “A Survey of Canadians’ Views on Open Science in Biobanking.” The online questionnaire as it appeared when hosted on the website SurveyMonkey.
(PDF)

S2 File Deidentified Questionnaire Responses. The complete exported survey data, with identifying answers relating to name, location and contact information removed.
(XLSX)

S3 File Complete Tables of Open-Ended Answer Codes. Tables showing every category identified by the coders, including the one-member categories which were not included in the tables within the manuscript.
(DOCX)

S4 File Themes in Respondents’ Other Potential Concerns About Biobank Projects. Table showing the coded themes for the responses to concluding Question 19, which was not included into the body of the manuscript.
(DOCX)

S5 File Geographic Location of Survey Respondents within Canada. Table showing the geographic distribution of respondents by province.
(DOCX)
