# Supplementary material for: Fair Shares and Sharing Fairly: A Survey of Public Views on Open Science, Informed Consent and Participatory Research in Biobanking
Source: PLoS One. 2015 Jul 8;10(7):e0129893. doi: 10.1371/journal.pone.0129893 (PMC4495996; doi:10.1371/journal.pone.0129893)
Supplement: S1 File — The online questionnaire as it appeared when hosted on the website SurveyMonkey. (PDF) [file pone.0129893.s001.pdf]

A Survey of Canadians' Views on Open Science in Biobanking

Consent Form

Purpose

The purpose of this study is to examine the public's opinion of "open science" in biobanking and genetic databases. These terms refer to a recent research model where human tissue samples, blood samples, DNA and/or personal information are collected, stored and shared extensively between collaborating scientists for research purposes. The legal and ethical implications of this model have not received much focus in policy or in public debate. Therefore, we are interested in your views on consent, confidentiality, and the ownership of products arising from "open science". We hope this information will help to address concerns about the sharing of biological samples and health information and to inform the development of future studies about research governance.

Eligibility

Our goal is to survey Canadians aged 18 and over who have either provided tissue, blood and/or DNA samples for scientific research, or who would consider doing so in the future.

Risks and Benefits

Because we are collecting information about your opinions, we do not anticipate any physical, psychological or social risks to participants. Participants will receive compensation in the form of Ipsos' i-Say points, which may be redeemed for cash, gift cards or charity donations as described in the "Rewards" section of the Ipsos website.

Procedure and Withdrawal

The following questionnaire will take about 30 minutes to complete. There will be no penalty or loss of compensation if at any time or for any reason you skip one or more questions or withdraw from the study entirely. Your participation in this study is completely voluntary.

Confidentiality

Only the Principal Investigator and two research assistants will have access to survey data. Your identifying information will be kept confidential and separated from your questionnaire answers in order to protect your identity and make your answers anonymous. If you withdraw from the study after the results are anonymized, it will be impossible to remove your questionnaire answers from the others.

All questionnaire information will be destroyed five years after we collect the surveys. The information from your consent form will also be destroyed at that time unless you select on this consent form that you are interested in being re-contacted, and you participate in another one of our surveys during that five years. In this case, your identifying information will be destroyed after ten years instead.

No personal information about you will be used in any report produced from this study. Results of this study may be shared in open-access journals, at academic conferences, to the media, and/or in reports and presentations to our funders, Le Fonds de la recherche du Quebec - santé (FRQS). Research participants will also be given access to aggregated study results at http://www.genomicsandpolicy.org/.

Contact

If at any time you have questions about this study or your rights as a participant, please contact either:

Principal Investigator
Yann Joly
PhD (DCL), Ad.E.
McGill University
E-mail: yann.joly@mcgill.ca
Phone: 514-398-4829

Study Coordinator
Derek So
MSc Human Genetics Candidate
McGill University
E-mail: derek.so@mail.mcgill.ca
Phone: 514-974-6721

If you wish to retain a copy of this form, please print this page now. Thank you for your time and effort.

*1. Please provide your name, mailing address, and email address.

Name:

Mailing Address:

E-mail Address:

***2. I am interested in being re-contacted for future studies about open science and genomic research.**

- ◯ Yes.
- ◯ No.

***3. By clicking "I agree", you indicate that you have read this consent form, that you are above 18 years in age, and that you consent to participate in this study. If you do not wish to participate in this study, please click "I disagree" or simply disregard this study instead.**

- ◯ I agree.
- ◯ I disagree.

Demographic Information

4. What is your age?

5. What is your gender?

- ◯ Female
- ◯ Male

6. What is the highest level of education you have completed? (If currently enrolled, please select the previous level or highest degree received.)

7. Have you provided tissue samples, blood samples, or genetic data to a biobank or genetic database research project? If not, would you consider doing so in the future?

- ◯ I have provided tissue samples or genetic data to a biobank or genetic database.
- ◯ I have not provided tissue samples or genetic data to a biobank or genetic database, but I might in the future.
- ◯ I have not provided tissue samples or genetic data to a biobank or genetic database, and I will not in the future.

Informed Consent

8. How important is it for you to receive clear, specific information about the objectives of any biobank or genetic database project you participate in?

◯ Very important ◯ Important ◯ Somewhat important ◯ Unimportant

9. How important is it for you to receive clear, specific information about the governance structure of any biobank or genetic database project you participate in (i.e. who will be responsible for managing your information and what safeguards are in place to protect the research participants)?

- ◯ Very important
- ◯ Important
- ◯ Somewhat important
- ◯ Unimportant

10. How important is it for you to receive clear, specific information about who will control the samples and data, and who could profit economically from any biobank or genetic database project you participate in?

- ◯ Very important
- ◯ Important
- ◯ Somewhat important
- ◯ Unimportant

11. How important is it for you to receive clear, specific information about how your confidentiality will be protected by any biobank or genetic database project you participate in?

- ◯ Very important
- ◯ Important
- ◯ Somewhat important
- ◯ Unimportant

12. What is the most important piece of information you would like to be provided about any biobank project that you participate in?

Data and Sample Sharing

13. What would you prefer concerning tissue samples from your body if they are left over after a medical procedure?

- ◯ I would prefer that my leftover tissues are not used except for my medical care.
- ◯ I would prefer to give a one-time general consent, which would allow the use of my leftover tissues in any type of medical research in the future.
- ◯ I would prefer that my leftover tissues are not used for medical research in general. I would be willing to give consent for their use in a specific area of research (e.g. 'cancer research', 'genetic research').
- ◯ I would prefer that my leftover tissues are not used in medical research unless I am re-contacted to give consent for each specific research project.
- ◯ Undecided.

14. Open sharing of genetic information between researchers and institutions has been shown to substantially facilitate health research. However, some parties are concerned that this could limit patients' rights to choose how, and by whom, their genetic information is used.

Would you be willing to participate in a project where your information and samples would be shared with the international community (without your name or personally identifying information attached), or would you prefer to participate in a project in which your data was only used by a single Canadian institution?

- I would prefer my data to be shared with the international scientific community.
- I would prefer my data to remain stored and used by a single Canadian institution.
- Undecided.

Control and Responsibility

15. Who should benefit financially from large-scale biobank research using genetic data and samples?

- The people who provided the samples.
- The community to which the people who provided the samples identify (e.g. all cancer patients, if the person who provided the sample had cancer).
- The researchers who have conducted the biobank project or their institution.
- Everybody.
- Undecided.
- Other (please specify).

16. Who should control and take ethical and legal responsibility for the genetic samples and data in a large-scale biobank or genetic database project?

- The people who provided the samples.
- The researchers who conducted the biobank project.
- The institution of the researchers who conduct the biobank project.
- Nobody should have control over genetic samples or data.
- Undecided.
- Other (please specify).

Confidentiality

17. How important is it that the identity and medical records of people who provide samples and data to a biobank or genetic database project are protected from access by third parties?

○ Very important ○ Important ○ Somewhat important ○ Unimportant

18. What risks, if any, would you associate with a breach of confidentiality in an open biobanking or genetic database research project?

Conclusion

19. Other than the topics discussed above, do you foresee any additional issues or concerns that open, collaborative biobanking or genetic database research could create?
