# Supplementary material for: Fair Shares and Sharing Fairly: A Survey of Public Views on Open Science, Informed Consent and Participatory Research in Biobanking
Source: PLoS One. 2015 Jul 8;10(7):e0129893. doi: 10.1371/journal.pone.0129893 (PMC4495996; doi:10.1371/journal.pone.0129893)
Supplement: S3 File — Tables showing every category identified by the coders, including the one-member categories which were not included in the tables within the manuscript. (DOCX) [file pone.0129893.s003.docx]

**Table A. Themes in Respondents’ Desired Information About Biobank Projects.**

| Themes | n | % |
| --- | --- | --- |
| project objectives | 43 | 47 |
| confidentiality | 19 | 21 |
| data protection | 18 | 18 |
| data management | 15 | 16 |
| return of results | 13 | 14 |
| relevance to donor | 9 | 10 |
| health applications | 8 | 9 |
| who benefits | 7 | 8 |
| use of profits | 6 | 7 |
| who has access | 6 | 7 |
| project governance | 5 | 5 |
| protection from discrimination | 5 | 5 |
| use for ethical purposes | 5 | 5 |
| protection from insurers | 4 | 4 |
| adherence to specific consent | 2 | 2 |
| destruction of data | 2 | 2 |
| protection from government | 2 | 2 |
| use after project completion | 2 | 2 |
| duration of use | 1 | 1 |
| financial compensation | 1 | 1 |
| funding source | 1 | 1 |
| government certification | 1 | 1 |
| how to contact for assistance | 1 | 1 |
| overall summary | 1 | 1 |
| project structure | 1 | 1 |
| project transparency | 1 | 1 |
| protection from employers | 1 | 1 |
| safety of donation process | 1 | 1 |
| use by foreign countries | 1 | 1 |
| use for common good | 1 | 1 |
| use of stem cells | 1 | 1 |
| use only for science | 1 | 1 |
| use to improve quality of life | 1 | 1 |

**Table B. Themes in the Risks Respondents Identified with a Confidentiality Breach.**

| Themes | n | % |
| --- | --- | --- |
| use by third party | 39 | 60 |
| identification of donor | 35 | 54 |
| use by corporations | 17 | 26 |
| insurance discrimination | 16 | 25 |
| disclosure of genetic risks | 11 | 17 |
| disclosure of personal information | 10 | 15 |
| identity theft | 9 | 14 |
| use in other research | 8 | 12 |
| employment discrimination | 6 | 9 |
| information released to general public | 6 | 9 |
| breach of trust | 5 | 8 |
| stigma | 5 | 8 |
| disclosure of medical records | 5 | 8 |
| contact by third party | 4 | 6 |
| use for profit | 3 | 5 |
| human cloning | 3 | 5 |
| effects on family | 3 | 5 |
| use by government | 2 | 3 |
| future research inhibited | 2 | 3 |
| misinterpretation | 2 | 3 |
| use for warfare | 1 | 2 |
| bioterrorism | 1 | 2 |
| misuse of resources | 1 | 2 |
| loss of individuality | 1 | 2 |
| disclosure of nonpaternity | 1 | 2 |
| safety hazards | 1 | 2 |
| loss of property | 1 | 2 |
| use by organ vendors | 1 | 2 |
| unknowable | 1 | 2 |
| use by banks | 1 | 2 |
| loss of information | 1 | 2 |
| human genetic engineering | 1 | 2 |
