# Supplementary material for: Fair Shares and Sharing Fairly: A Survey of Public Views on Open Science, Informed Consent and Participatory Research in Biobanking
Source: PLoS One. 2015 Jul 8;10(7):e0129893. doi: 10.1371/journal.pone.0129893 (PMC4495996; doi:10.1371/journal.pone.0129893)
Supplement: S4 File — Table showing the coded themes for the responses to concluding Question 19, which was not included into the body of the manuscript. (DOCX) [file pone.0129893.s004.docx]

**Table S4. Themes in Respondents’ Other Potential Concerns About Biobank Projects.**

| **Themes** | **n** | **%** |
| --- | --- | --- |
| use for profit | 8 | 27 |
| use for unethical purposes | 7 | 23 |
| loss of privacy | 4 | 13 |
| loss of control of samples | 4 | 13 |
| use by government | 3 | 10 |
| human cloning | 2 | 7 |
| leak of information | 2 | 7 |
| mismanagement | 2 | 7 |
| patenting | 2 | 7 |
| restriction of results | 2 | 7 |
| use by corporations | 2 | 7 |
| ageism | 1 | 3 |
| eugenics | 1 | 3 |
| geneticization | 1 | 3 |
| identity theft | 1 | 3 |
| information added to medical records | 1 | 3 |
| insufficient funding | 1 | 3 |
| lack of accountability | 1 | 3 |
| lack of public knowledge | 1 | 3 |
| misregulation | 1 | 3 |
| racial discrimination | 1 | 3 |
| theft of data | 1 | 3 |
| use by fertility clinics | 1 | 3 |
| use for warfare | 1 | 3 |
| use without consent | 1 | 3 |
