# Supplementary material for: Fair Shares and Sharing Fairly: A Survey of Public Views on Open Science, Informed Consent and Participatory Research in Biobanking
Source: PLoS One. 2015 Jul 8;10(7):e0129893. doi: 10.1371/journal.pone.0129893 (PMC4495996; doi:10.1371/journal.pone.0129893)
Supplement: S5 File — Table showing the geographic distribution of respondents by province. (DOCX) [file pone.0129893.s005.docx]

**Table S5. Geographical Location of Survey Respondents within Canada.**

| Province of Residence | n | % |
| --- | --- | --- |
| Overall | 114 |  |
| British Columbia | 16 | 14 |
| Alberta | 8 | 7 |
| Saskatchewan | 2 | 2 |
| Manitoba | 4 | 4 |
| Ontario | 46 | 40 |
| Québec | 11 | 10 |
| New Brunswick | 3 | 3 |
| Prince Edward Island | 1 | 1 |
| Nova Scotia | 4 | 4 |
| Newfoundland and Labrador | 3 | 3 |
| do not wish to specify | 16 | 14 |
